# Consumer Neuroscience-Based Metrics Predict Recall, Liking and Viewing Rates in Online Advertising

**DOI:** 10.3389/fpsyg.2017.01808

**Published:** 2017-10-31

**Authors:** Jaime Guixeres, Enrique Bigné, Jose M. Ausín Azofra, Mariano Alcañiz Raya, Adrián Colomer Granero, Félix Fuentes Hurtado, Valery Naranjo Ornedo

**Affiliations:** ^1^Instituto de Investigación e Innovación en Bioingeniería, Universidad Politécnica de València, València, Spain; ^2^Departamento de Comercialización e Investigación de Mercados, Facultad de Economía, Universitat de València, València, Spain

**Keywords:** neuromarketing, YouTube, artificial neural networks, eye tracking, heart rate variability, brain response

## Abstract

The purpose of the present study is to investigate whether the effectiveness of a new ad on digital channels (YouTube) can be predicted by using neural networks and neuroscience-based metrics (brain response, heart rate variability and eye tracking). Neurophysiological records from 35 participants were exposed to 8 relevant TV Super Bowl commercials. Correlations between neurophysiological-based metrics, ad recall, ad liking, the ACE metrix score and the number of views on YouTube during a year were investigated. Our findings suggest a significant correlation between neuroscience metrics and self-reported of ad effectiveness and the direct number of views on the YouTube channel. In addition, and using an artificial neural network based on neuroscience metrics, the model classifies (82.9% of average accuracy) and estimate the number of online views (mean error of 0.199). The results highlight the validity of neuromarketing-based techniques for predicting the success of advertising responses. Practitioners can consider the proposed methodology at the design stages of advertising content, thus enhancing advertising effectiveness. The study pioneers the use of neurophysiological methods in predicting advertising success in a digital context. This is the first article that has examined whether these measures could actually be used for predicting views for advertising on YouTube.

## Introduction

Advertising effectiveness is still challenging academics and practitioners. Neuroimaging and physiological measurement tools are becoming popular within marketing (Daugherty et al., 2016). Their primary uses are related to unconscious measures based on eye movement, heart rate and brain activity, among others (see Venkatraman et al., 2015 for more details). Such tools aim to provide better understanding of the impact of affect and cognition on memory (Vecchiato et al., 2013). Furthermore, neurophysiological methods can capture the dynamics of television commercials content because they provide continuous data unlike traditional measures, such as interviews and surveys that only reflect a global indicator for every commercial.

Global expenditure on media has been rising over the years and digital advertising is the fastest-growing category (McKinsey, 2015). Marketers are still calling for accurate assessments about advertisement effectiveness and about the return on advertising expenditure (McAlister et al., 2016). Nowadays, Internet advertising has evolved dramatically and a platform like YouTube is a good example on how to reach viewers at a global scale.

Advertisers pursue the attention of viewers and seek ad recall, brand recall and positive emotions. If this occurs, ads will be stored in the viewers’ long-term memory. Humans may not remember each advertisement they have been exposed to, but neuroscience techniques can detect conditions that lead to the memorization of advertising.

The usage of neuro metrics in measuring advertising effectiveness overcome some of the weaknesses associated with traditional measures (Varan et al., 2015). Despite the benefits of using such measurements, the key question is the choosing of the variables of advertising effectiveness. Among the different types of effects pursue by advertising (see Moriarty et al., 2012), three types of effects have been considered. First, perception, and particularly exposure to the ad is recognized as the first step in any evaluation process. In this study, we adopt the online views as the measure of advertising effectiveness of online ads; secondly, the emotional dimension is typically used in evaluating the effects of advertising, thus we adopt the liking as an emotional metric; lastly, the cognition effect of advertising is measured through ad recall.

Neurophysiological methods offer richer data than self-report measurements of particular interest in advertising research. Firstly, physiological measurements of emotion allow researchers to analyze emotional activity without cognitive bias. Secondly, neurophysiological methods provide instant and continuous data that allow researchers to decompose the data analysis into small pieces of study. Lastly, physiological measurements typically offer a myriad of metrics. In the present study several metrics are compared and two new metrics derived from eye tracking (ET) are also proposed: “number of quadrants per second” (Quad_sec) and “gaze brand effectiveness ratio” (Brand_ratio). However, physiological measures have their own limitations: a strong reliance on physiological data to measure emotions leaves room for misinterpretation of physiological noise (e.g., natural changes in body status) and burdens researchers with the difficult task of attributing specific physiological changes (e.g., increase in heart rate) to complex and subjectively experienced emotions (e.g., hate, love, or fear).

Scholar research has recently adopted neurophysiological measures to better understand consumer responses to advertising (Astolfi et al., 2009). To the best of our knowledge, no articles in marketing have previously examined whether these measures could actually be transferred into real life views on advertising on YouTube. The work by Venkatraman et al. (2015) was one of the first pieces of research that tried to establish correlations between brand performance and physiological responses whilst viewers watched ads. So far it has been hard to gauge the number of viewers who will watch an ad, which in turn is one of the main objectives for marketers. However, digital platforms overcome this situation enabling researchers to measure consumer unconscious reactions to ads and the number of views.

Neurophysiological methods to measure advertising effectiveness are becoming popular including consolidated tools such as ET and facial reader (Wedel and Pieters, 2014), EGG (Ohme et al., 2010) and more recently sophisticated tools such as fMRI (Venkatraman et al., 2015; Couwenberg et al., 2017). New extensions to sales are emerging, Thus, biomarkers can, to some extent, predict sales figures (see Kühn et al., 2016) and even in virtual reality experiments have been developed (see Bigné et al., 2016).

This paper aims to answer whether neurophysiological methods contribute anything beyond traditional methods in predicting ad success in a digital context. Specific research goals are listed below. Firstly, to analyze whether three of the most cited neurophysiological and behavioral techniques: electroencephalogram (EEG), heart rate variability (HRV) and ET correlate with common cognitive states typically used in advertising research (e.g., liking and recall measures) (Morin, 2011; Ruanguttamanun, 2014; dos Santos et al., 2015). Secondly, we aim to explain whether the variance in the number of views on a brand’s official YouTube channel is related to any of the neurophysiological measures and their metrics.

A study to assess subjects’ responses to nine 30-s online television ads was conducted. Data gathered from 47 subjects were split into six datasets based on three conditions: recall (RMB) vs. no-recall (FRG), liking (LIKE vs. DISLIKE) and Internet views (>5M vs. <5M). All the metrics extracted from physiological and behavioral responses were compared and correlated between these groups.

The contributions of this paper are listed hereafter. First, we show how different metrics from three neurophysiological devices correlate in an attempt to select the most accurate ones for digital commercials. Second, Artificial Neural Networks (ANN), using biometric data can predict digital views of ads, hence common physiological patterns related to unconscious responses can predict when an ad is going to be remembered or liked. Finally, two new metrics are proposed to measure advertising effectiveness in digital advertising, which show a high level of accuracy in predicting digital views. In this paper, we build on the later works that reviewed Super Bowl ads with ET and heart rate (Christoforou et al., 2015), and brain response (Deitz et al., 2016), but adding for the first time joined in neural networks models, three of the most employed signals in advertisement research (EEG, HRV and ET) and proposing two novel metrics based only on eye-tracking data that predicts viewer’s preferences in video advertisements.

The rest of the paper is organized as follows. Firstly, we provide a brief literature review of advertising research effectiveness and we introduce neurophysiological methods related to ad recall and ad likeability. Secondly, we describe the experimental design and signal recording and processing techniques used to extract biometric data. Then, we describe the study results in three parts. In the first part, a comparison of the metrics from neurophysiological signals related to likeability of the ad and ad recall. In the second one, the correlations between these biometrics, the score given by participants in a poll (e.g., ACE_score) and the number of views on YouTube are exposed; and thirdly, by applying ANN to these biometric datasets, we predict the number of views on YouTube for each ad tested during the study. Finally, we discuss the contributions and implications for researchers and practitioners.

### Established Methods in Advertising Research

Despite the diverse approaches used in advertising research (Vakratsas and Ambler, 1999), advertising success on ad execution has focused on traditional measures such as liking, excitability, and recall (Venkatraman et al., 2015). Acknowledging the established literature (Astolfi et al., 2009; Kim et al., 2014), this paper focuses on liking and recall as traditional measures. Online polls have been adopted in academic research as a valuable data source (see Strach et al., 2015). Based on a US national-representative Internet sample of 500 respondents, Ace Metrix has been providing advertising effectiveness scores since January 2009 and it is used in this study.

### Advertising Research in a Digital Setting

Digital channels have changed advertising research dramatically and, as a result, a new paradigm is emerging (Ha, 2008; Bigné, 2016). One of the major gains is that analytics are available at ad level, including exposure measured through number of views and likeability through “likes.”

### Neurophysiological Tools in Advertising Research

This study focuses on three neurophysiological methods, aiming to collect data from different angles: eye movements, heart variability and brain responses.

Eye tracking is a well-established measure of visual attention (Wedel and Pieters, 2014; Venkatraman et al., 2015) to different stimuli, such as product choice (Guerreiro et al., 2015), static images (Mould et al., 2012), printed ads (Elsen et al., 2016) banner ads (Lee and Ahn, 2012) and videos of the Super Bowl (Christoforou et al., 2015).

Heart rate variability is the physiological phenomenon of variation in the time interval between heartbeats. It is measured by the variation in the beat-to-beat interval (Task Force of the European Society of Cardiology and The North American Society of Pacing and Electrophysiology, 1996). This variability of the heart is related to activations of the sympathetic and parasympathetic systems of the autonomic nervous system. HRV provides an independent measure of attention (Lang et al., 1999) and it has been applied to television commercials (Acharya et al., 2006; Grandjean et al., 2008; Geisler et al., 2010; Bellman et al., 2013; Valenza et al., 2014; Venkatraman et al., 2015).

Electroencephalogram is an electrophysiological monitoring method to record the electrical activity of the brain. The relationship between affection, engagement and brain activation in frontal brain activity has been well documented in psychology and neuroscience research (Harmon-Jones et al., 2010; Khushaba et al., 2013). Emotional frontal asymmetry as hypothesized by Davidson (2004) has been applied to analyze commercials (Ohme et al., 2010; Vecchiato et al., 2011), including Super Bowl ads (Deitz et al., 2016) and advertising success (Venkatraman et al., 2015). Vecchiato et al. (2011, p. 582) showed that “activity” in the left-frontal cortex related to “pleasant” commercials and activity in the right-frontal cortex associated with “unpleasant” commercials.

### Hypothesis Development

As briefly discussed earlier, ET, HRV and EEG provide measures of responses to advertising stimuli and might be related to ad performance. This study attempts to examine the relationship between three types of data from neurophysiological tools, ET, HRV and EEG, and three advertising variables typically used in advertising research, such as ad recall, ad likeability and ad views. Hypotheses will be anchored in three streams of research aiming to integrate them into a single approach: (i) theoretical advertising literature; (ii) online advertising; (iii) neurophysiological research related to advertising. An integrative approach is useful because neurophysiological primary data *per se* are non-meaningful for advertising research. Therefore, this type of data must be interpreted in relation to classic advertising assumptions in order to prove their validity. Most of the data gathered in this type of studies are based on a different methodological paradigm that derives from psychophysiology (Bolls et al., 2012).

1. Advertising research shows a positive relationship between recognition and attention. Donthu et al. (1993) found a positive relationship between attention and recall for outdoor advertising. In an ET study, Pieters et al. (2002) found that attention led to ad recognition. Baack et al. (2008) posit that recognition is considered an immediate measure of attention.
2. As discussed earlier, ET and some metrics from EEG can capture attention, hence if attention leads to recall, it could be argued that each neurophysiological tool can capture attention.

The Advertising Research Foundation’s Copy Research Validity Project (CRVP) showed in the early nineties that advertising likability is the single best measure of effectiveness (Rossiter and Eagleson, 1994). Furthermore, likeability has been considered relevant and important in measuring commercial effectiveness in the ads aired in the Super Bowl, showing stable scores between 1990 and 1999 (Tomkovick et al., 2001). The positive influence of likeability has also been highlighted recently in online settings. Thus, likeability of online video ads has successfully linked to intention to share them (Shehu et al., 2016), which can be interpreted as a successful performance. Recent literature in neuromarketing also highlights a relationship between liking and HRV, ET measurements and fMRI signals (Venkatraman et al., 2015).

Online video platforms, such as YouTube, have been largely approached from the user-generated content perspective (see Smith et al., 2012). However, its dimension as a digital channel for watching commercials has almost been neglected, with some related exceptions (Verhellen et al., 2013). The number of views of each online video, including commercials, is available on YouTube, and it is commonly seen as a valid measure of its popularity. Given its social media nature, recent research is addressing two main fields of interest: the sources that drive views to a video and the preferred type of content. A recent study by Zhou et al. (2016) identified YouTube search and related video recommendation as the major view sources. In adopting YouTube views, the age of the video and the potential replays must be considered. Research shows that user’s preference seems relatively insensitive to the video’s age (Cha et al., 2007). More recently, Chen et al. (2014) analyzed a lifetime model of online video popularity that features the following three main characteristics of interest here, adopting views as a potential variable in explaining our intended relationships: (i) views follow a Zipf distribution; (ii) replay percentage is very low; (iii) and only video content on news and sports are strongly dependent on age, with popularity being much less sensitive to age in music videos, which can be considered closer to ads. Therefore, views can be adopted as a valid measure over time of ad exposure.

Based on previous reasoning on recall, likeability and number of views, and their relationship with neurophysiological tools, therefore:

- H1: Eye tracking, HRV and brain activity capture (a) recalled and non-recalled online ads, (b) liked and disliked online ads, and (c) ads with high numbers vs. low numbers of YouTube views.

1. Online polls (e.g., Ace Metrix, YouGov) are becoming popular in the industry and have been used for academic purposes. Established scores in the market are considered by marketers as a valuable source of information. Ace Metrix is a company that tests ads within 24–48 h of each ad’s initial airing and provides meaningful scores. Ace Metrix component scores range between 1 and 950. Scores for the general population are normally distributed and approximately centered around 530. For obtaining Ace Score, every ad is shown to a unique set of 500 respondents who complete standardized surveys that assess the ad both quantitatively and qualitatively providing rich insights by demographic segment. All ads are scored on 6 factors most likely to influence consumer behavior including relevance, likeability, information, change, attention and desire—as well as re-watch ability, purchase intent and brand linkage. After respondent scores are collected, Ace Metrix component scores are computed and assigned to each ad, creating an overall score called the Ace Score. Since its inception, Ace Metrix has consistently used the same methodology to measure the effectiveness of every ad they have tested. As a result, they are in a unique position in the field of advertising effectiveness to assess relative advertising performance between any competitive set of ads imaginable, both across different industries and different time periods.
2. In addition, the digital world is changing data availability and triggering new analytical research methods where new research avenues for unstructured data, including neurodata, and new methods like ANN call for new research (Wedel and Kannan, 2016). Venkatraman et al. (2015) conducted a similar study but in the offline context. They found that deceleration correlated with liking (*r* = 0.37, *p* < -05) and recognition (*r* = 0.34, *p* < 0.05). The current study differs in two ways: (i) we parse neurophysiological correlations in a different context, such as digital exposure featured by searching rather and displaying; (ii) Venkatraman et al. (2015) captured advertising effort, through GRPs, and advertising outcome, through advertising elasticities; however, our study attempts to find out the correlates of neurophysiological metrics from ET, HRV and EEG with independent variables of ad effectiveness based on both, survey data and digital views. Therefore, we predicted:

- H2: Eye tracking, HRV and brain activity capture correlate with (a) self-reported score of ad effectiveness and (b) online views on YouTube.

As stated before, YouTube as a digital channel to watch commercials has almost been neglected with some related exceptions (Verhellen et al., 2013). Our aim here is to use ANN to predict the number of online views of ads placed in YouTube, where the input variables are metrics from ET, HRV, and EEG. ANN is useful for parsing non-linear relationships and adopt feed forward and back propagation approaches (West et al., 1997). ANN has been successfully applied in advertising since the mid-nineties (Curry and Moutinho, 1993). Research posits the superiority of these methods over other statistical approaches. Surprisingly, this is the first attempt to use ANN to predict online views. In our study, we aim to classify and to predict online views based on metrics from ET, HRV, and EEG. Therefore, we predicted:

- H3: Artificial neural networks based on mixed data from ET, HRV and brain activity predict the number of online views on YouTube.

## Materials and Methods

### Participants and Design

Final sample consisted of 35 randomly healthy volunteers (15 women and 20 men, mean age = 25; *SD* = 5 years) recruited from the city where the lab is located. Initial sample measured was 47 subjects but after an examination of the dataset was carried out, 12 participants were removed due to corrupted data from experimental sessions in some of the acquired signals. All of the participants showed corrected-to-normal vision and hearing. They were asked to pay attention to the documentary as in a common situation. No mention of the importance of the ads was made. The study was approved by the Institutional Review Board of the *Polytechnic University of Valencia* with written informed consent from all subjects in accordance with the Declaration of Helsinki.

**FIGURE 1:**
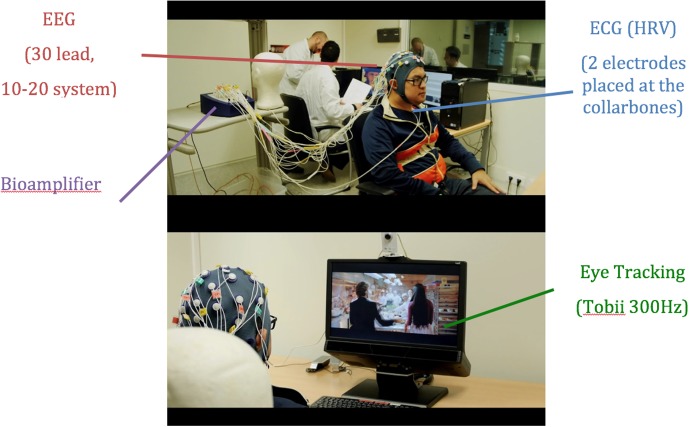
Participant in the study. (**Top**: The EEG cap is visible. ECG electrodes placed on the chest and TMSI equipment). (**Bottom**: Eye tracking equipment is shown).

**FIGURE 2:**
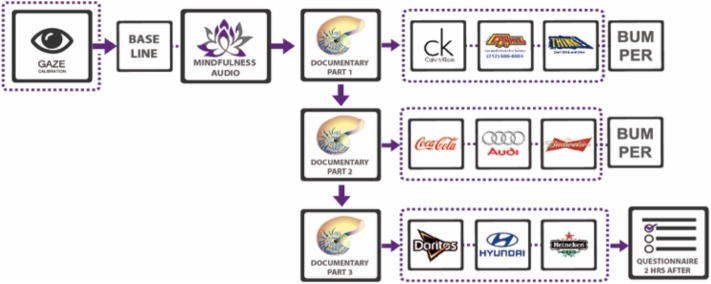
Stimuli and experimental design.

The experiment was conducted in a neuromarketing lab of a large European university and comprises the three parts shown in **Figure 1**. In Parts 1 and 2, participants sat comfortably on a reclining chair with a 32-channel EEG device, with two electrodes to measure heart variability and an eye-tracker (**Figure 1**). In Part one, participants were exposed to a mindfulness audio designed by experts to help them relax and disconnect from past experiences of the day (Fjorback et al., 2011; Demarzo et al., 2014). Then, in Part two they were shown a 30-min long documentary with three commercial breaks of three ads lasting about 30 s each; the first break occurred after 7 min, the second in the middle of the documentary, and the third 7 min before the end as **Figure 2** depicts. At the end of this second part, participants were informed that an interview would be held 2 h later (Part 3).

### Pretesting and Stimuli

Television commercials from the final of the Super Bowl 2015 were chosen because they are a good representation of the most searched high impact ads. We wait for a year to get results of number of views on the brand’s official YouTube channel and a final selection of eight from 47 ads was made to represent a uniform distribution of ads ranked by number of views and also to represent a distribution of different commercial products (**Table 1**). In addition to the number of views, the ACE Metrix^1^ score was also obtained for some ads. The ACE Metrix is the most employed US scale drawn up by consumers that evaluates the ad creative effectiveness based on viewer’s reaction to national TV ads. The results are presented on a scale of 1–950. The selected television commercials belong to international brands of commercial products such as drinks (3), food (1), cars (2), textiles (1) and services (1). None of the ads had been broadcasted in the country where the experiment was performed in order to remove previously unchecked exposure of the subjects to the proposed stimuli. These videos were randomly distributed during the sessions with participants to avoid bias in data analysis.

**Table 1:** ACE metrix score, number of visits on a brand’s official YouTube channel during a year and ranking of visits established for the selected video advertisements regarding number of visits.

	ACE_score	No. visits	Rank of visits^∗^
Drink 2	665	12,037,414	D
Car 2	394	10,583,315	D
Drink 3	Non-evaluated	10,356,789	D
Drink 1	641	6,652,069	C
Food	626	4,594,507	B
Textile	362	2,952,014	B
Car 1	611	1,453,022	B
Service	167	15,340	A


*^∗^VISIT_RANK: A: = <1M views; B = 1M–5M views; C = 5M–10M views; D = >10M views.*

### Data Recording and Processing

#### Cerebral Recording (EEG)

Electrical activity of the brain was recorded by a stationary 32-channel system (REFA 32, TMSI hardware). EEG activity was gathered at a sampling rate of 256 Hz. The experiment used 30 Ag/AgCl water-based electrodes and bracelets attached to the opposite wrist of the subjects’ dominant hand. The montage of brain electrodes followed the international 10–20 system (Jasper, 1958).

The EEG baseline was removed and channels detected as having corrupted data were rejected and interpolated from the closest electrodes (Colomer Granero et al., 2016). When a channel with erroneous data was identified, kurtosis was employed computing the fourth standardized moment in the signal of each electrode. This kurtosis is defined in Equation 1

$$K(x)=\frac{\mu_{4}}{\sigma_{4}}=\frac{E\left[{\left(x-\mu \right)}^{4}\right]}{{E\left[{\left(x-\mu \right)}^{2}\right]}^{2}}$$

where μ_4_ was the fourth moment of the mean, σ was the standard deviation and E[x] was the expected value of signal x. The EEG signal was segmented in one second acquired accordingly to the experiment events. The intra-channel kurtosis level of each epoch was used to reject the epochs with high levels of noise.

To detect artifacts from eye movements, blinking and muscular activation, Independent Component Analysis (ICA) (Gao et al., 2010) and automatic method (ADJUST) (Mognon et al., 2011) were implemented. Each EEG artifact-free trace was band pass filtered twice in order to isolate only the spectral components in delta (1–3 Hz), theta (4–7 Hz), alpha (8–12 Hz), beta (13–24 Hz), beta extended (13–40 Hz) and gamma bands (25–60 Hz).

To quantify the cerebral activity in each band, the Global Field Power (GFP) (Wackermann et al., 1993) was calculated as explained in previous work (Colomer Granero et al., 2016).

Recent studies have shown that the main areas involved in the phenomena of memorization and pleasantness are the frontal areas (Astolfi et al., 2009). For that reason, calculation of electrodes in frontal lobe were taken into account. A GFP signal was then calculated for each frequency band considered in the experiment. GFP associated with each ad analyzed and GFP during a period taken as the baseline of watching a 2-min neutral documentary before the block of ads were compared and normalized to obtain the corresponding *z-score* index.

In addition to the *z*-score of GFP for each EEG band, two metrics applied in advertising research were also calculated: The *Pleasantness Index (PI)*, and the *Interest Index (II).* The PI is a metric calculated over time that provides information about the pleasantness of the stimuli presented (Vecchiato et al., 2011). Brain activity gathered by left-frontal electrodes is compared with brain activity registered by the right-frontal electrodes (frontal asymmetry). These comparisons are made with GFP in the theta and alpha bands, comparing asymmetric pairs of electrodes.

The questionnaire generated the likeability score for each ad under study. Using this information, participants were segmented into two groups: “LIKE” and “DISLIKE.” Then, the brain Pleasantness Index, PI was calculated for each group as describes (Colomer Granero et al., 2016).

The II, enables an advertising evaluation of user interest in theta and beta bands (Vecchiato et al., 2010). The most relevant peaks of these signals are selected. Two parameters were obtained: the number of peaks during a particular ad (PN_total_) and the number of peaks during the periods the brand name appeared in that particular ad (PN_brand_). Accordingly, the II was calculated as describes (Colomer Granero et al., 2016).

#### Heart Rate Variability

To analyze HRV, the electrocardiogram, ECG signal needs to be filtered (Blanco-Velasco et al., 2008), analyzed to detect QRS zones (Pan and Tompkins, 1985) and revised manually by an expert, because the appearance of a single ectopic can produce variations in certain key parameters extracted from this analysis (Clifford, 2006). HRV analysis can generate a set of metrics that can be extracted from different dimensions: time, frequency, time-frequency and non-linear. Parameters extracted from the time domain used in this study were: average heart rate (t_meanHR), standard deviation of continuous HR values (t_sdHR), the square root of the sum of successive differences between adjacent RR intervals (t_RMSSD) and the number of successive pairs of RR intervals showing a difference of more than 50 ms between them (t_NNx).

Power spectral density (PSD) analysis provides information about the amount of power in the frequency bands defined for the beat-to-beat interval signal generated. In this case, we employed the Lomb-Scargley method (Castiglioni and Di Rienzo, 1996). The frequency bands defined (low frequency, LF and high frequency, HF) are stated by Task Force of the European Society of Cardiology and The North American Society of Pacing and Electrophysiology (1996). Power metrics can be presented in absolute values (aLF, aHF, aTotal), normalized to total energy (nLF, nHF) or in a percentage value of total energy (pLF, pHF). The ratio established between the LF and HF band provided information on the sympathetic/parasympathetic balance. The power value of the peak in the fundamental frequency (peakLF, peakHF) was also extracted.

Non-linear analysis was run using techniques such as the Poincaré graph that give us SD1 and SD2 metrics (Fishman et al., 2012). Sample entropy (sampen) is another non-linear technique that attempts to quantify the complexity or degree of new information generated (Richman and Moorman, 2000). If entropy is equal to zero, then consecutive sequences are identical. Similarly, bigger values show higher complexity of the analyzed signal.

To summarize, for each ad analyzed by subject, HRV metrics were computed by means of a computational analysis plug-in based on Matlab (Guixeres et al., 2014). For the purpose of this study, the seventeen most relevant metrics employed in HRV analysis (Task Force of the European Society of Cardiology and The North American Society of Pacing and Electrophysiology, 1996) were selected based on time, frequency and non-linear domains.

#### Eye Tracking

The Tobii TX300 eye tracker^2^ was used in this experiment as **Figure 1** depicts. This eye-tracker collects gaze data at 300 Hz. The subsequent analysis of raw data used Tobii Studio 3.2 software. For each commercial the following metrics were obtained from the gaze data: (i) number of fixations during each ad (Fix_Count_Advert); (ii) average duration of fixations during an ad (Fix_Dur_Advert); Furthermore, we obtained several metrics from the times the brand appeared in each ad. To calculate such metrics, a dynamic Area of Interest (AOI) that followed the brand was created using TOBII studio software to obtain (iii) the average duration of fixations exclusively focused on the brand (Fix_Dur_Br); (iv) the number of fixations during the brand appearance that focused on it (Fix_Count_Br); (v) the time from the appearance of the brand until it was fixated on for the first time (FFIX_Dur_Br); (vi) the number of visits inside the brand’s AOI (Visit_Count_Br); and (vii) average duration of visits to the brand’s AOI (Visit_Dur_Br). For the last two metrics, it should be remembered that a visit is the event that starts when the eye enters an AOI until it leaves such AOI. In addition to these metrics, two new metrics are proposed in this study: (viii) Number of Quadrants per second (Quad_sec); and (ix) the Gaze Brand Effectiveness Ratio (Brand_ratio).

Quad_sec enables the way the user explores a space with his eyes to be quantified. To calculate this metric, the screen surface was divided into a grid of 4 × 4 equal-sized quadrants. Then, the average quantity of different quadrants that the eye visited per second was calculated for each commercial. Higher values for this metric meant that the subject explored the space in “ambient mode,” covering all the space with his eyes, whilst lower values meant that the subject explored the space in “focus mode,” centering his visual attention on specific zones. These two modes of watching an image stimulus have been reported in previous works (Bradley et al., 2011; Holmqvist et al., 2011).

$${Quad}_{seg}=\frac{N_{q}}{t_{s}}$$

where N_q_ is the number of visits to quadrants during stimulus presentation and t_s_ is the duration in seconds of the stimulus.

Brand_ratio enables the effectiveness of visual attention toward the brand during the ad to be quantified. To calculate Brand_ratio, brand appearance was controlled by setting an AOI around the brand every time it appeared in the commercial. Then this metric was defined as the number of seconds that the subject looked directly at the brand divided by the total time that the brand was present on the screen during the commercial. This metric could be related to the participant’s interest in and familiarity with the brand as it relates to the time that eye and brain are able to identify a brand, a concept related to familiarity (Kent and Allen, 1994).

$${Brand}_{ratio}=\frac{t_{bf}}{t_{b}}$$

where t_bf_ is the time in seconds that the gaze fixed on the brand and t_b_ is the total time in seconds that the brand appeared during the ad.

#### Questionnaire

Data were sorted using three criteria: (i) spontaneous ad recall; (ii) ad liking; (iii) the number of online views was used as a control variable. The criterion for ad recall was to remember without clues brand names of the commercials 2 h after the study. Accordingly, participants were split into two subgroups. The first dataset was related to the biometric activity collected during the observation of the recalled commercials 2 h after being exposed to them. This dataset was named RMB. The second subset included the biometric activity collected during observation of the non-recalled commercials, (FRG). The ad liking criterion was related to the biometric activity collected during observation of the television commercials that the subjects rated 5 or above on a 10-point Likert scale, being this subset named LIKE and DISLIKE, respectively.

### Statistical Methods

In order to test comparisons of means of the metrics calculated, a set of Shapiro-Wilk tests (W) were conducted to test whether dependent variables deviated from normality. Then a statistical analysis was carried out using ANOVA for metrics with normal distribution and the Mann Whitney non-parametric test for metrics that did not show a normal distribution. A corrected *p*-value less than *p* = 0.005 was chosen to correct multiple comparisons effect (Feise, 2002).

To get the correlation of neurometrics with the ACE score and the number of online views of the ads on YouTube, a Pearson correlation was applied to the number of visits so it could be considered as a linear scale. However, Spearman’s correlation was applied to the ACE score so it could be considered as a rank variable instead of a linear scale.

#### Artificial Neural Networks

We adjusted two neural networks with SPSS statistics using all metrics defined for EEG, HRV and ET, and including gender, as our input variables. The first network was adjusted to classify advertising responses into a ranking for the number of views on YouTube (RANK_VISITS). This ranking divided ads into four clusters (<1M: ads with less than 1 million visits, 1M–5M, 5M–10M, and >10M: ads with more than 10 million visits). The second network was adjusted to predict the real number of visits for each ad.

Two kinds of network were compared with the same data. Multi-layer Perceptron networks (MLP) and Radial Basis Function networks (RBF). Regarding accuracy for classification and estimation of the data, MLP networks were selected finally for the two purposes instead of RBF. After testing results changing several parameters in MLP architecture, a final structure was chosen for the both neural networks (see Appendix). To validate accuracy of networks, cross-validation technique was employed. Entire sample was divided into two groups (70% of cases for training the network and 30% of cases to assess classification accuracy) and that validation was repeated 10 times (*k* = 10), selecting each time different groups for training and assessing. Final results were averaged from the 10 turns.

## Results

### Biometric Mean Comparison

In order to test H1, we conducted a comparison among means of the metrics calculated from EEG, HRV and ET. As stated earlier, these metrics were compared by means of the following two conditions, recall vs. non-recall after 2 h (RMB vs. FRG) and by likeability (LIKE vs. DISLIKE).

### Brain Response Comparison

**FIGURE 3:**
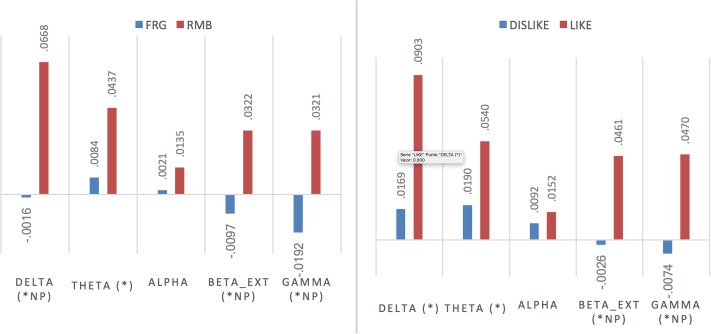
Results from brain response (EEG) comparing GFP results for 5 specific bands (^∗^values with significant differences *p* < 0.005; NP, non-parametric test carried out).

**Figure 3** shows the comparison among means for *z*-score indexes in each frequency band for the different factors chosen. In the case of remembered ads, the RMB group shows significant differences compared to the FRG group with higher values in the delta, theta, beta ext. and gamma bands. The LIKE group showed significant differences compared to the DISLIKE group with higher values in the delta, theta, beta ext. and gamma bands.

In the pleasant index (PI) and II, there were no significant differences in the comparison between the recalled and liked groups.

### HRV Response Comparison

**Table 2** shows the comparison of means for HRV metrics for the different factors chosen. In the case of the remembered ads, the RMB group showed significant differences compared to the FRG group, with higher values in the non-linear SD2 Poincaré index (p_SD2) that reflect higher continuous beat-to-beat variability (Piskorski and Guzik, 2007). The LIKE group showed significant differences compared to the DISLIKE group with higher values in the energy of the low frequency band (f_aLF_lomb) which is associated with sympathetic activation (Task Force of the European Society of Cardiology and The North American Society of Pacing and Electrophysiology, 1996).

**Table 2:** Mean comparisons of HRV metrics in the time, frequency and non-linear domains.

	RMB		FRG			UNLIKE		LIKE		
						
HRV Metrics	Mean	*SD*	Mean	*SD*	*p*	Mean	*SD*	Mean	*SD*	*p*
t_meanHR	71.9	12.3	70.6	8		69.6	8.8	71.1	7.8	
t_sdHR	5.2	7.3	5.5	4.5		5.1	5.6	5.3	2.7	
t_NN50	13.3	18.4	13.8	12.7		13	13.6	13.5	13.3	
t_RMSSD	43.5	32.3	54.6	112.3		43.2	26	60.1	142.7	
f_aLF_lomb	0.031	0.015	0.033	0.013		0.031	0.014	0.035	0.014	(^∗^NP)
f_aHF_lomb	0.027	0.020	0.027	0.017		0.029	0.021	0.026	0.017	
f_aTotal_lomb	0.060	0.027	0.061	0.022		0.062	0.028	0.062	0.024	
f_nLF_lomb	0.539	0.223	0.573	0.2		0.547	0.203	0.594	0.194	
f_nHF_lomb	0.461	0.223	0.427	0.2		0.453	0.203	0.406	0.194	
f_LFHF_lomb	2.022	2.156	2.121	2.057		1.786	1.545	2.412	2.412	
f_peakLF_lomb	0.1	0.02	0.09	0.02		0.09	0.02	0.09	0.02	
f_peakHF_lomb	0.27	0.07	0.26	0.07		0.26	0.07	0.25	0.07	
p_SD1	30.7	22.8	32.8	21.4		30	18	32.9	19.8	
p_SD2	66.1	28	77.6	36.7	(^∗^NP)	68.8	30.4	80.3	38	
p_SD1vsSD2	0.497	0.382	0.441	0.192		0.462	0.224	0.423	0.154	
nl_sampen1	2.175	0.227	2.141	0.243		2.179	0.25	2.143	0.217	
nl_sampen2	1.803	0.486	4.19	0.532	(^∗^NP)	9.373	0.788	1.66	0.389	


*^∗^Values with significant differences *p* < 0.005; NP, non-parametric test.*

### ET Response Comparison

**Table 3** shows the comparison of means for eye-tracking metrics for the different classic metrics and the two-new metrics proposed. In the case of the recalled ads, the RMB group showed significant differences vs. the FRG group, generating lower values in (Visit_Dur_Br). The LIKE group did not show significant differences compared to the DISLIKE group.

**Table 3:** Mean comparisons of ET metrics.

	RMB		FRG			UNLIKE		LIKE		
						
Eye Tracking Metrics	Mean	*SD*	Mean	*SD*	*p*	Mean	*SD*	Mean	*SD*	*p*
Brand_ratio	0.36	0.25	0.32	0.25		0.37	0.26	0.31	0.24	
Visit_Count_Br	6.7	2.42	6.65	2.33		7.29	2.53	6.32	2.38	
Visit_Dur_Br	0.76	0.24	0.63	0.25	(^∗^NP)	0.75	0.23	0.62	0.38	
FFix_Dur_Br	0.18	0.14	0.16	0.13		0.17	0.13	0.15	0.13	
Fix_Dur_Br	0.18	0.12	0.17	0.11		0.18	0.15	0.17	0.12	
Fix_Count_Br	28.23	28.97	23.73	22.87		33.76	37.48	21.98	19.29	
Quad_sec	2.47	1.35	2.48	1.29		2.65	1.62	2.58	1.24	
Fix_Dur_Advert	0.24	0.1	0.24	0.11		0.24	0.12	0.21	0.11	
Fix_Count_Advert	273.32	120.34	281.8	200.12		340.73	180.73	270.83	165.43	


*^∗^Values with significant differences *p* < 0.005; NP, non-parametric test.*

Hypothesis 1 is confirmed, as there are significant differences in each signal (EEG, HRV and ET) between ads (i) recalled and non-recalled (22% of comparisons) and (ii) liked and disliked (16% of comparisons).

As regards H2, centered on the correlation of neurometrics with the ACE score and the number of online views of the ads on YouTube, 19 of the 25 metrics showed significant (*p* < 0.01) correlation with the ACE score and 15 of the 25-metrics showed significant correlation with the number of online views. In EEG, the *z*-score in the delta band correlated with both indexes and the *z*-score in the theta band correlated with the ACE score. Both indexes, pleasantness and interest, showed high values in terms of significant correlation with the ACE score. In particular, there was a high level of correlation between PI_theta and the number of visits. In terms of ET, the proposed metric (Brand_ratio) showed significant correlations with both indexes, PI and II. In addition, Quad_sec showed significant correlation with the ACE_score. Visit duration and Fixation count on the brand showed significant correlation with the number of visits. Fixation duration showed a negative correlation with the ACE score and the value of the correlations of the Fixation count during the ad for both indexes was relevant. Focusing on HRV, t_NN50 showed significant correlations with both indexes. The total energy band; LF and HF bands, also showed correlations with both indexes. The normalized values of LF and HF and the sympathovagal index (LFHF) showed significant correlations with the ACE score. Frequency with the maximum peak on the HF band (peakHF) and the SD2 Poincaré index also showed significant correlations with the number of visits. The type 1 non-linear parameter sample entropy index (sampen1) showed significant correlations with both indexes whilst sampen2 only correlated with number of visits. Therefore, H2 is confirmed, showing significant correlations between metrics for EEG, HRV and ET with ACE score and number of visits on YouTube.

### Predicting Ad Effectiveness on the Internet with ANN

#### ANN to Rank Visit Classification

Two kinds of ANNs were compared with the same data: MLP and RBF. In terms of accuracy when classifying data, MLP networks worked better than RBF. After testing results and changing several parameters in the MLP architecture, the final structure chosen had one hidden layer with a tangent hyperbolic activation function and an output layer with a softmax activation function. All the co-variables were typified (see Appendix for more details).

**Table 4** shows the final results for the classification of the training and the test dataset using the neural network. The percentage of correct predictions in the test dataset was 82.9%. Three higher ranking levels (1M–5M, 5M–10M, and >10M) showed high effectiveness ratios of classification (88.5, 100, and 90%, respectively). Only the first group (<1M) showed a poor effectiveness ratio of 36.4%, mistaking more than 54% of cases for second level (1M–5M).

**Table 4:** Results for the successful classification of training and test dataset applying an adjusted neural network.

Classification					

	Real rank order	Predicted				
		
		<1M	1M–5M	5M–10M	>10M	% succ.
Training	<1M	17	1	0	0	94.4%
	1M–5M	0	60	0	0	100.0%
	5M–10M	0	0	23	1	95.8%
	>10M	0	0	0	67	100.0%
	Overall percentage	10.1%	36.1%	13.6%	40.2%	98.8%
Test	<1M	4	6	1	0	36.4%
	1M–5M	2	23	0	1	88.5%
	5M–10M	0	0	9	0	100.0%
	>10M	0	0	3	27	90.0%
	Overall percentage	7.9%	38.2%	17.1%	36.8%	82.9%


*Number of cases in each possible class and percentage of successful cases.*

Of the 37 input variables used in neural networks, the importance of each normalized metric to predict was extracted from the neural network. This metric shows the most relevant variables for classifying each case according to the correct ranking. The pleasantness index extracted from the theta band (PI_theta, 100%) was the most important parameter for brain metrics, followed by II_theta (56.6%) and PI_ alpha (56.5%). Regarding HRV metrics, the mean of HR (64.20%) was the most representative parameter, followed by SD1 Poincaré (61.40%) and t_RMSSD (58.30%). In terms of eye-tracking metrics, the fixation count during the ads (59.30%) was the most important index, followed by the average fixation count during brand appearance (59.30%), and the average duration of fixations during brand appearance (45.30%). Gender inclusion and whether the ad was consciously remembered came last in the ranking with very little importance. The proposed metrics from ET showed medium importance (Ratio_Brand: 40.80% and Quad_sec: 40.20%). When comparing the frequency brain bands, the theta band behaved best, in the PI index (100%), II index (56.6%) and *z*-score (37.50%).

### ANN for Estimating the Number of Online Views

As described above, MLP and RBF were compared only in terms of accuracy (time execution was extremely low in both cases). MLP networks also worked better than RBF networks in terms of accuracy when estimating the number of visits. After testing results and changing several parameters in the MLP architecture, the final structure chosen had one hidden layer with a tangent hyperbolic activation function and an output layer with an identity activation function. All the co-variables and the dependent variable were typified.

For the final MLP structure, the first subset was used to train the MLP and the estimated values of the number of visits were obtained from the second subset to test the accuracy of the network. Final results for the estimation of training and the test dataset using an ANN. Relative error from the test dataset was 0.199, that is, a significant level of variance.

Of the 37 input variables used in the neural networks, the pleasantness index extracted from the theta band (PI_Theta, 100%) was again the most relevant variable in the neural network in predicting the number of views. Regarding brain metrics, PI_theta was followed by PI_alpha (13.10%) and II_theta (11.60%), though these were a long way behind. In HRV metrics, sample entropy (53.70%) was the most important parameter, followed by the total energy of frequency band (43.40%) and t_RMSSD (41.70%). Regarding eye-tracking metrics, the count of fixations during ads (44.30%) was the most important index, as on the first occasion, followed by number of visits during brand appearance (27%) and then followed very closely by the count of fixations during brand appearance (26.60%). The inclusion of gender in the model and whether the ad was consciously recalled ranked last and do not improve prediction. The proposed new metrics extracted from ET showed different results. Quad_sec showed a medium-low importance (16%) and Ratio_Brand a low importance (6.70%). Half the HRV metrics were in the upper positions.

**FIGURE 4:**
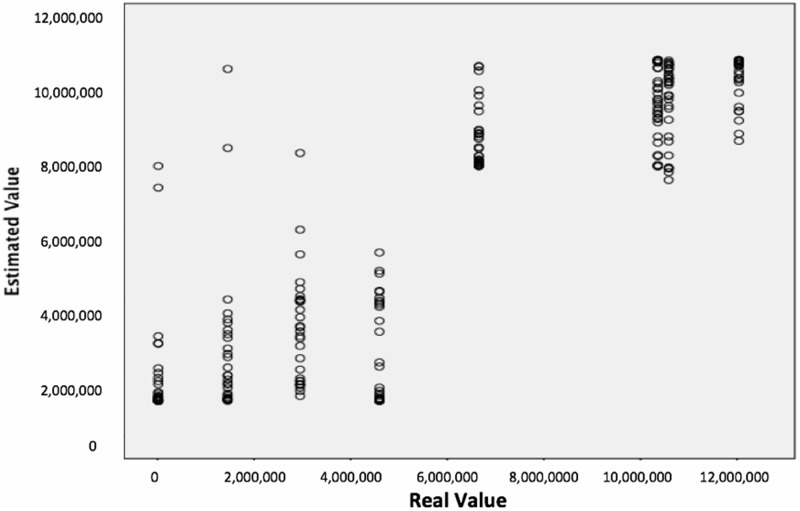
Scatterplot showing predicted vs. observed-values for number of visits.

**Figure 4** represents predicted vs. observed values as a scatterplot for each case. Figure shows that neural network classifier worked well in classifying real data regarding biometric response, specifically observing the cluster of ads with higher number of views vs. ads with poor audience. There was some dispersion in all the ads in terms of real values.

## Discussion

In this study, several hypotheses have been confirmed. The first hypothesis was supported, showing significant differences in physiological responses (EEG and HRV) and ET for each of the three dimensions raised (i.e., recall, liking and visits). In cases where participants remembered the ad after 2 h, results showed higher probability that the spectral amplitude in the RMB condition was always higher than the power spectra in the FRG conditions (Astolfi et al., 2008). A statistical increase of PSD in the prefrontal and parietal areas for the RMB dataset compared with the FRG dataset was in line with the suggested role of these regions during the transfer of sensory perceptions from short-term memory to long-term memory storage. Specifically, there were higher values in the theta band for the cases where the ad was remembered, which is in line with other studies (Werkle-Bergner et al., 2006; Boksem and Smidts, 2014; Vecchiato et al., 2014). Regarding this condition, an interesting future analysis should look at the biometric response differences between people that remember the ad without remembering the brand or vice versa.

In terms of the HRV analysis, the means of the sample entropy parameters were significant, showing the higher complexity of heart variability in cases where the ad was remembered. Valenza et al. (2012), showed that Approximate Entropy, decreased during arousal elicitation using images from International Affective Picture Session (IAPS) but there are no studies until now that have related HRV entropy with remember cognitive function.

In ET, cases where ads were unrecalled showed longer duration of visits to the brand when it appeared on screen. Higher values for this metric are related to difficulty in identifying an object (Goldberg et al., 2002; Wedel, 2013). This fact could be explained by poor identification of the brand, which could indicate that the ad will be forgotten in the short term.

Regarding cases where participants rated the ad positively or negatively, brain activity was stronger in terms of PSD in the LIKE group than in the DISLIKE group. These results are congruent with another EEG studio based on the observation of pictures from the international affective picture system (Aftanas et al., 2004). In HRV, there were only significant differences in energy in the LF frequency band associated with sympathetic activation. In ET, no significant differences were found for the proposed metrics.

The second hypothesis was supported showing significant correlations between physiological and eye-tracking responses with the ACE score and with the number of online visits. All the indexes (pleasantness and interest) calculated showed significant correlations. The pleasantness index in the theta band presented an especially high correlation with the number of visits and the ACE score. In ET, there were relevant correlations with both metrics proposed in this work. Brand_ratio showed a positive linear relation between the percentage of time watching a brand and the number of views on the Internet. The number of fixations for the brand showed a positive relation with the number of views on the Internet. Regarding HRV, there was a positive correlation with tNN50 for both outputs. All the energy values in the frequency bands (total, LF and HF) were negatively correlated with both outputs. The ACE score was positively related to the normalized LF band associated with sympathetic activation. Non-linear entropy parameters also showed a negative correlation with both outputs revealing that an increase in the complexity of the HRV signal is associated with less quality and effectiveness of the ad on the Internet.

Hypothesis three aimed to test whether ANN with relevant biometrics could represent an interesting technique to classify ads based on their ranking on the Internet and to estimate the number of visits on the Internet. The results obtained showed that the ANN were able to accurately classify and estimate the effectiveness of each ad on the Internet via their biometric response. The results for the first network, which were adjusted to classify each ad based on a four-level ranking, showed a global average accuracy of 82.9%. Poor accuracy was obtained with ads with a lower number of views but this could have been improved if more ads in this ranking had been selected in the stimulus group. The relevant metrics for this classification were the pleasantness index and II in the theta band, the mean heart rate and the SD1 Poincare in HRV, the number of fixations during the ad and inside the brand for ET. Results for the second network to estimate the number of views on the YouTube for each ad showed a relative error of 0.199. The most important metrics for this estimate were the pleasantness index in the theta band, entropy in HRV and the number of fixations during advertising in ET. Despite good results to estimate the number of views, it would seem that classifying ads according to a ranking constitutes a better approach, taking into account the excellent results obtained in the first classifier.

Further research is needed, with more studies comparing new techniques for classification, such as Linear Discriminant Analysis, Marquardt Backpropagation Algorithm, and Deep Learning. In addition, new metrics and new signals extracted from biometric responses must be tested to find out which parameters are best to evaluate the effectiveness of advertising. The group of different categories of advertising must also be increased. Future studies should also focus on adjusting personalized classifiers to advertising categories (fashion, food, social, etc.), different channels (e.g., Facebook) and formats (desktop or mobile). Also, new metrics like facial gesture coding (McDuff et al., 2015) and fNIRS (Kopton and Kenning, 2014) could be mixed in new models for predicting Ad effectiveness.

## Conclusion

This study has shown that aspects related to the impact of advertising, such as whether the ad is going to be remembered or whether it is going to be highly rated can be detected from an analysis of consumers’ biometric responses during the viewing of these ads. We also found differences in the impact of advertising in terms of gender, which encourages the use of these biometric data to design advertising content that is tailored to each individual group of population. Other variables, such as age, cultural level, and even personality could be explored in future studies to test whether there are similar differences to the ones found in gender. The final conclusion that this study has yielded is that the effectiveness of a new ad on YouTube can be predicted using metrics extracted from EEG, HRV and ET. Up until now, there has been no evidence that biometric responses can help to classify the numbers of views on YouTube for an ad. This study has also contributed with two new metrics for ET that can be used in research on advertising. These results will help to explain the success of advertising responses showing an interesting methodology to be use by practitioners designing advertising content.

## Author Contributions

JG is the corresponding author. JG, EB, and MAR designed the study. JAA conducted the study. JG, EB, and JAA conducted the literature review and wrote the research summaries. ACG and VNO analyzed the EEG data, JP and JAA analyzed HRV and ET data. MAR and EB are the directors of this work. JG wrote the first draft of the manuscript, and all authors contributed to and have approved the final manuscript. Authors had full access to the study data.

## Conflict of Interest Statement

The authors declare that the research was conducted in the absence of any commercial or financial relationships that could be construed as a potential conflict of interest.

## Footnotes

1 http://www.acemetrix.com/

2 Tobii.com
