# Supplementary material for: Consumer Neuroscience-Based Metrics Predict Recall, Liking and Viewing Rates in Online Advertising
Source: Front Psychol. 2017 Oct 31;8:1808. doi: 10.3389/fpsyg.2017.01808 (PMC5671759; doi:10.3389/fpsyg.2017.01808)
Supplement: Supplementary file 1 [file Data_Sheet_1.DOCX]

Appendix A

Spearman and Pearson correlations between biometrics (EEG, HRV y Eye Tracking) and ACE score (s) and number of views (p) on official YouTube channel. (bold values are significant at 0.01 level (2-tailed))

| **ACE score** | **Number of visits** |  |
| --- | --- | --- |
| s | p |  |
| ***Brain response (9 metrics)*** |  |  |
| *LOG_ZSCORE.Delta* | **0.216** | **-0.160** |
| *LOG_ZSCORE.Thetha* | **0.175** | -0.078 |
| *LOG_ZSCORE.Alfa*  *LOG_ZSCORE.BetaEXT* | 0.078 | -0.076 |
|  | 0.083 | 0.015 |
| *LOG_ZSCORE.Gamma* | 0.061 | -0.011 |
| *Pleasant Index_Thetha* | **0.527** | **0.792** |
| *Pleasant Index_Alpha* | **0.520** | 0.042 |
| *Interest Index_Thetha* | **0.333** | **0.173** |
| *Interest Index_BetaExt* | **0.424** | **0.241** |
| ***Eye Tracking (9 metrics)*** |  |  |
| *Brand_ratio* | **0.191** | **0.279** |
| *Visit Count_MARCA_Mean* | -0.010 | -0.028 |
| *Visit Duration_MARCA_Mean* | 0.130 | **0.196** |
| *First Fixation Duration_MARCA_Mean* | 0.130 | -0.064 |
| *Fixation Duration_MARCA_Mean* | 0.074 | -0.101 |
| *Fixation Count_MARCA_Mean* | 0.040 | **0.249** |
| *Quadrants by second* | **0.141** | -0.038 |
| *Fixation Duration_ANUNCIO_Mean* | **-0.136** | -0.035 |
| Fixation Count_ANUNCIO_Mean | **0.473** | **0.450** |
| ***Heart Rate Variability (17 metrics)*** |  |  |
| t_meanHR | -0.022 | 0.029 |
| t_sdHR | 0.062 | 0.097 |
| t_NN50 | **0.266** | **0.346** |
| t_RMSSD | 0.009 | 0.076 |
| f_aLF_lomb | **-0.156** | **-0.287** |
| f_aHF_lomb | **-0.232** | **-0.280** |
| f_aTotal_lomb | **-0.289** | **-0.375** |
| f_nLF_lomb | **0.143** | 0.098 |
| f_nHF_lomb | **-0.143** | -0.098 |
| f_LFHF_lomb | **0.143** | 0.010 |
| f_peakLF_lomb | 0.036 | 0.002 |
| f_peakHF_lomb | -0.120 | **-0.141** |
| p_SD1 | 0.016 | 0.062 |
| p_SD2 | 0.135 | **0.159** |
| p_SD1vsSD2 | -0.117 | -0.089 |
| nl_sampen1 | **-0.152** | **-0.163** |
| nl_sampen2 | **-0.152** | -0.087 |

Parameter and final structure chosen for the neural network related to classify Ads regarding input data into RANK_VISIT.

| **Perceptron Multilayer Neural network (MLP)** | | | |
| --- | --- | --- | --- |
| Input Layer | Number of units | | 39 |
|  | Method to adapt scale in covariables | | Typificated |
| Hidden Layer | Number of hidden layers | | 1 |
|  | Number of units of hidden layer | | 5 |
|  | Activation function | | Tangent hyperbolic |
| Output Layer | Dependent variables | 1 | RANK_VISIT |
|  | Number of units | | 4 |
|  | Activation function | | Softmax |
|  | Error function | | Cross Entropy |

Normalized importance index for the main parameters employed as input at the neural network to classify AD regarding input data into RANK_VISIT (red: EEG metrics, blue: HRV metrics, green: ET metrics, purple: questionnaire metrics).

Parameter and final structure chosen for the neural network related to estimate Number of Visits for each Ad in YouTube regarding biometric data.

| **Information about perceptron multilayer neural network** | | | |
| --- | --- | --- | --- |
| Input Layer | Number of units | | 39 |
|  | Method to adapt scale in covariables | | Typificated |
| Hidden Layer | Number of hidden layers | | 1 |
|  | Number of units of hidden layer | | 2 |
|  | Activation function | | Tangent hyperbolic |
| Output Layer | Dependent variables | 1 | VISIT |
|  | Number of units | | 1 |
|  | Method to adapt scale in dependent variable | | Typificated |
|  | Activation function | | Identity |
|  | Error function | | Sum of squares |

Normalized importance index for the main parameters employed as input at the neural network to predict number of visits (red: EEG metrics, blue: HRV metrics, green: ET metrics, purple: questionnaire metrics).

| **Results of the artificial neural network** | | |
| --- | --- | --- |
| Training | Sum of squared error | 15.283 |
|  | Relative Error | 0.173 |
| Test | Sum of squared error | 6.509 |
|  | Relative Error | **0.199** |

Relative error results for predicting the number of views on an official YouTube channel
